# Development and characterization of 17 polymorphic microsatellite markers for the reef manta ray (*Mobula alfredi*)

**DOI:** 10.1186/s13104-019-4270-8

**Published:** 2019-04-22

**Authors:** Amelia J. Armstrong, Christine L. Dudgeon, Carlos Bustamante, Michael B. Bennett, Jennifer R. Ovenden

**Affiliations:** 1 School of Biomedical Science, The University of Queensland, Brisbane, Australia; 2 Facultad de Recursos Naturales Renovables, Universidad Arturo Prat, Iquique, Chile

**Keywords:** Population genetics, Conservation genetics, Microsatellite loci, Short tandem repeats

## Abstract

**Objective:**

Limited sample sizes are often a problem for species of conservation concern when using genetic tools to make population assessments. Lack of analytical power from small sample sizes can be compensated for by use of a large marker set. Here we report on development and characterization of 17 novel microsatellite markers for the reef manta ray (*Mobula alfredi*).

**Results:**

Loci were screened on 60 reef manta rays (*M. alfredi*) sampled from the east coast of Australia. The number of alleles per locus varied from 2 to 13 with observed heterozygosities ranging between 0.300 and 0.917. The development of these 17 additional markers increases the total number of microsatellite markers available for this species to 27.

## Introduction

The reef manta ray (*Mobula alfredi*) occurs in tropical and subtropical waters of the Indian and Pacific oceans [1]. The species forms aggregations in coastal waters throughout its range, which provides for targeted ecotourism in many coastal regions [2]. However, these aggregations may suffer depletion from competing by-catch and targeted fisheries, particularly for their highly-valued body parts that are used in ‘traditional medicines’ [3–6]. Like many large shark and ray species, *M. alfredi* is long-lived with conservative life history parameters (i.e. slow growth, late maturity, low fecundity) that make populations highly-susceptible to over-exploitation [7]. As such, the global conservation concerns for this species have resulted in its classification as ‘Vulnerable to Extinction’ on the IUCN Red List [8], and has led to listing in Appendix II of the Convention on the International Trade in Endangered Species and Appendices I and II of the Convention on Migratory Species. Future research priorities identified for reef manta rays include investigation of population structure (regional and global) and connectivity, based on genetic analyses [9].

To date, the limited genetic work conducted on DNA from reef manta rays has been primarily in the context of broader phylogenetic studies [10, 11]. One recent phylogenetic study resulted in the collapse of the genus *Manta* into *Mobula*, and a reassignment of the position of *M. alfredi* within this genus [12]. An earlier study used nuclear DNA obtained from *M. alfredi* sampled in Japanese waters to develop ten species specific microsatellite markers [13]. However, preliminary studies on of *M. alfredi* from the east coast of Australia found that only eight of the previously developed markers were polymorphic, with the remaining markers either non-polymorphic or non-amplifiable. Collection of adequate sample sizes to overcome the limitation of few markers is often difficult, expensive and inappropriate for species of conservation concern. Therefore, we address this issue through the development of additional microsatellite markers for *M. alfredi* that may allow for robust estimation of population sizes without the need to sample large numbers of animals, and will facilitate future investigations into fine-scale structure (e.g. relatedness).

For studies focused on non-model species of conservation concern, microsatellite markers can be amplified from relatively small quantities of DNA and provide a relatively low cost per sample for genotyping (especially when working with pre-developed marker sets). While genotype-by-sequencing approaches (i.e. RADseq, DArTseq) are increasingly used for genomic applications to non-model species, the strict requirements surrounding DNA quality, quantity and contamination may limit application to historical, degraded or small amounts of sampled-DNA. Furthermore, microsatellite markers provide the potential for a standardised marker set that can facilitate comparisons of genetically distinct populations across broad temporal and geographical scales. Here we report the development of 17 polymorphic microsatellite markers for *M. alfredi*, to augment the 10 previously developed, and detail marker performance through the genotyping of 60 individual manta rays from the east coast of Australia. We anticipate that microsatellite markers developed here will inform ongoing regional studies on population connectivity, and may facilitate global population comparisons through the provision of an enlarged and published marker set.

## Main text

### Methods and results

Microsatellite primer sequences were developed de novo from DNA obtained from a Mobula alfredi voucher specimen from Lady Elliot Island Reef, Australia. A genomic library was developed with the TruSeq Nano DNA sample prep kit (Illumina, San Diego, CA) and sequenced using an Illumina HiSeq 2000 platform (San Diego, CA) that supported the acquisition of 2 × 125 base pair (bp) paired-end reads, following the protocols supplied by the manufacturer. Sequences between 150 and 400 bp were explored for microsatellite motifs using the software QDD v.3.1 [14] following the microsatellite discovery protocol described by Vargas-Caro et al. [15]. In brief, perfect microsatellite repeat motifs with > 10 repeating units were identified and loci with product sizes estimated at less than 100 bp were removed. Additional loci where flanking regions contained ‘runs’ (of a single base) or repeated sequence elements were excluded. Selected sequences consisting of repeat motifs and flanking sequences were blasted against NCBI GenBank using default parameters to exclude loci potentially located in coding regions. Primers with homology outside the target flanking region or that may amplify more than one locus were excluded by blasting back against the genomic library using GENEIOUS v 9.1.8 (http://www.geneious.com) [16]. Of the remaining candidate primer pairs, we selected 48 to take through to development, giving priority to loci with tri or tetra nucleotide repeat motifs. For the 48 candidate microsatellite loci developed, a ‘CAG-tail’ was attached to the 5′ end of the forward primers allowing downstream use of fluorescent PCR product labelling. Loci were combined in multiplexed PCRs to avoid overlapping product sizes. The 5′ end of the reverse primer had a ‘GTTT-tail’ attached to ensure complete adenylation, thereby protecting PCR products from non-template nucleotide addition and facilitating accurate genotyping [17].

Genotyping was conducted using genomic DNA extracted from ethanol preserved tissue biopsies collected non-lethally (2015–2018) from *M. alfredi* in waters surrounding Lady Elliot Island (− 24.11°, 152.71°) and North Stradbroke Island (− 27.42°, 153.54°), Queensland, Australia. Possible sample duplication was avoided, as each manta ray’s unique ventral skin pattern was photographed at the time of biopsy collection [18]. Loci were amplified in a 12 µl PCR reaction comprised of 1–2 µl of genomic DNA (10–15 ng), 0.5 µl primer stock, 6 µl of 2 × MyTaq mix (Bioline Australia), with remaining volume made up to 12 µl with Milli-Q H_2_O. PCR cycling conditions were a 95 °C for 60 s step, followed by 38 cycles of 95 °C for 60 s, 95 °C for 15 s, 55 °C for 15 s and 72 °C for 15 s, with a final extension step of 72 °C for 15 min. The diluted PCR products (1:10 dilution) were denatured with formamide and an internal size standard (Genescan 500-LIZ) was added. Fragment separation was performed with an ABI 3730 DNA Analyser. Allele scoring was undertaken using the GENEIOUS Microsatellite Plugin Version 1.4 (GENEIOUS v 9.1.8) [16]. Loci were initially amplified in singleplex reaction against 6–12 individuals. Loci that successfully amplified were characterised against an additional 18–24 individuals to determine information content before selection for inclusion in a multiplex PCR. After removing loci that either did not amplify, produced an inconsistent product, or were mono-morphic, the 48 candidate loci were reduced down to a final set of 17 (Table 1).

**Table 1:** Summary details for 17 microsatellite loci developed for *Mobula alfredi*

Locus	NCBI reference	Repeat motif	Primer sequence (5′-3′)	Fluoro label	*n*	A	Observed allele size range (bp)	H_O_	H_E_	*P*_HWE_
MAQDD04	MK225654	GAA_(27)_	F: ACATCCTGCAGAGGCTGTAA R: TCCTTATCTTGTTCCGGCAG	VIC	60	10	363–393	0.88	0.82	0.71
MAQDD09	MK225655	TG_(15)_	F: CGTCGACTGTTCACACTTCC R: CTTCCTGGCTGGTAGGTACG	FAM	60	5	190–206	0.62	0.59	0.59
MAQDD11	MK225656	TC_(19)_	F: TTTACCTATAGATGGGTCAGGGAC R: GCAGATCGCTGTAGCTGTGA	NED	60	8	204–224	0.92	0.85	0.07
MAQDD12	MK225657	AC_(19)_	F: AAAGGATCTGGGAATACGGG R: GTGTTGGGATCTCTGGTGCT	FAM	60	4	149–155	0.32	0.36	0.48
MAQDD18	MK225658	TG_(13)_	F: GGGAGACATTGAAACGTGTG R: GAATGTGATGGAGAGCACTTG	FAM	60	3	137–149	0.30	0.38	0.11
MAQDD21	MK225659	TG_(13)_	F: GGCATACAAAGTGCTGGAGG R: ATCTCAACCCTGCGTGGAC	NED	60	13	270–314	0.65	0.69	0.24
MAQDD22	MK225660	AC_(17)_	F: CGGAATAGCTGACCCTCAGA R: ATTGTGATGGAGTGGTCCGT	FAM	60	3	134–138	0.68	0.55	0.18
MAQDD23	MK225661	AC_(10)_	F: CCCATCACTGATAATGAGCGT R: GTGCAGTCAAAGGAATGGGT	FAM	60	2	148–150	0.35	0.50	0.02
MAQDD26	MK225662	AC_(16)_	F: ATGTCAACCGGACTGGAAAT R: TGTATGCGTATCTAGCATCTTCA	PET	60	6	203–33	0.62	0.59	0.08
MAQDD28	MK225663	AC_(23)_	F: GCTGCAGGGAAGAAGCTAAA R: TTTGTCCGAATATCTTGCCA	NED	60	6	151–165	0.58	0.55	0.95
MAQDD29	MK225664	GT_(21)_	F: GCTGAGAGGAAGCTATTGGC R: GAAGATACAGGAAGATAAATTGGCA	FAM	60	8	193–213	0.63	0.60	0.90
MAQDD31	MK225665	AC_(20)_	F: AACTGCCCATTCACATTTCC R: CCCATAGCTACCATCAGGGA	PET	60	13	282–314	0.72	0.72	0.34
MAQDD33	MK225666	CA_(21)_	F: GACGAGACATCCCAAACACC R: TTTCCTGGATCCACAACCTC	FAM	60	7	200–228	0.65	0.59	0.31
MAQDD37	MK225667	GT_(14)_	F: CGTTGCATCCATCTGACGTA R: GATGCACCAGAGCAACCAC	PET	60	4	170–192	0.58	0.51	0.36
MAQDD38	MK225668	CA_(14)_	F: CCAAACATAACCCAGGCAAG R: CAGCCTTGGTGAACTGAGTG	VIC	60	5	156–172	0.65	0.68	0.96
MAQDD39	MK225669	GT_(14)_	F: AGCAGCTCTGTGGAAGCAGT R: GCACCCATTTCCAATCAGTT	FAM	60	2	360–362	0.65	0.50	0.04
MAQDD43	MK225670	AC_(14)_	F: TCCCTGGCTGTATTGCCTAC R: AACCCTCGGATATCCATTCTC	NED	60	7	162–184	0.80	0.73	0.55

*NCBI* National Center for Biotechnology Information GenBank Reference; Primer Sequence *F* forward, *R* reverse (All forward primers had a GTT on the 3′ end & all reverse primers had CAG tag on the 5′ end (not included here), *n* is the number of individuals genotyped, A is the number of alleles observed, H_O_ is the observed heterozygosity, H_E_ is the expected heterozygosity, *P*_HWE_ is the probability of the locus deviating from Hardy–Weinberg equilibrium

A total of 60 *M. alfredi* from the Australian east coast population were genotyped with these 17 loci. To ensure there were no scoring errors or allelic dropout, all traces were checked using MICRO-CHECKER [19]. Observed and expected heterozygosities were calculated using GENALEX v 6.5 [20]. Locus MaQDD04 had a tri-nucleotide motif with the remaining markers exhibiting di-nucleotide repeats. The total number of alleles per locus ranged from 2 (MaQDD23) to 13 (MaQDD21 & MaQDD31). MaQDD04 displayed the highest observed heterozygosity (H_o_ = 0.88) with heterozygosity ranging widely for di-nucleotide loci (H_o_ = 0.35–0.80). Exact tests identified two loci (MaQDD23, *p *= 0.0212 & MaQDD39, *p *= 0.036) not in Hardy–Weinberg equilibrium, as calculated by GENEPOP v 4.2 [21]. Linkage disequilibrium tests (GENEPOP v4.2) revealed significant linkage between only one pair of loci (MaQDD04 & MaQDD11, *p *= 0.00), after Bonferroni correction.

### Conclusions

The need for high quality, standardized genetic markers resulted in the development of 17 novel microsatellite for reef manta rays. This marker set supplements microsatellite primers already available for the species [13], and enables research questions to be addressed that require the higher statistical power provided by a larger marker set. It is hoped that these additional markers also facilitate collaboration between studies of reef manta ray populations globally through the provision of named primers for which resultant genotypes and diversity metrics can be collaboratively compared and contrasted for reef manta ray populations globally. The combination of these novel markers with those already developed previously for reef manta rays [13] provides up to 27 markers for use in population structure and genetic effective population size investigations. The delineation of population structure and generation of population size estimates have been highlighted as research priorities to help guide conservation action for the reef manta ray [9].

## Limitations

At the time of primer development and manuscript preparation, the study did not have access to a sufficient number of samples from other locations for cross-population comparisons. Recent genotyping suggests marker applicability for *M. alfredi* sampled on Australia’s west coast (approximately 5600 km from east coast sampling locations) (author’s unpublished data). Due to sample availability and permit restrictions, this study did not test cross-species (within the family Mobulidae) primer amplification for the newly developed microsatellite loci.
